# Health and Psychology

**Published:** 1920-02-21

**Authors:** 


THE PUBLIC WELL-BEING—(*continued*).

### Health and Psychology.

The course of lectures at the Institute of Hygiene is rather a psychological series than a hygiene series, for in the bright addresses which have hitherto been delivered the lecturers have discussed the effects on health of new hats, the psychology of unrest, and the psychological results of war in various directions. Last week Mr. Grant Ramsay spoke under the title of "Hygiene as a Remedy for Social Unrest," and he proceeded to analyse social unrest under the two heads of Labour and Women's Emancipation "so-called." Both, he says, are moving too fast. It is likely that his views will be regarded as old-fashioned by most people engaged in either of those movements. But he also made drastic suggestions which must shock the old-fashioned.

There is sound common-sense in his emphasis on the fact that honest, steady labour brings contentment, and that idleness is an easy fomenter of unrest. There is also truth in his statement—with due recognition of the fact that many elements of labour would laugh it to scorn—that with better organisation, feeding, housing, and variety of work, there is no reason why any fit man should not work twelve hours a day. That would certainly be intensive labour, and would require to be tackled like a dinner—in courses—and it is admitted, on the other hand, that even six hours daily is too long for some forms of labour. But it is common knowledge that the longest days of labour and the happiest days are often those of men who, with variety of interests, work of their own free will for a regular number of hours which no trade union would countenance. It would be still more difficult to convince trade unionists that, if only they could see into the future, lower wages would be the slogan to-day, because they mean lower prices, less profits, better supplies, better quality, and better health. That is a proposition with ramifications which certainly need some economic thinking out.

Mr. Ramsay is rather severe on the women's movement, which, he declares, is marked less by emancipation than by climbing down to the level of men. Here, again, his economics are of a sweeping character. Many, he said, desired to save women from the strain of professional and commercial life, but, while the disproportion of the sexes remained, justice demanded that every vocation should be free and open to them. But where women were up against the laws of nature, nature was bound to win. The primary duty was maternity, and, if this were evaded, there was an end to all things.

He had investigated the question of the falling birth-rate in France thirty years ago, and had concluded that the chief cause affecting maternity was the psychological influence traceable to the degree in which French women shared their husbands' worries in business as well as in the home. Excitement and gaiety also adversely affected the birth-rate, and, although we were advised to take particular care of our baby boys, we must also see to our future mothers, who hold the destinies of the Empire in their lap.

And what are Mr. Ramsay's remedies? Hygiene, good food, temperance in all things, rationing of house accommodation as an offset against the supineness of the authorities on the housing problem, and, as some people would always create slums wherever they lived, a properly laid on supply of fresh air, under pressure, in every house in the same way as gas and fresh water are supplied. Bermondsey cannot always go to Brighton, but there is no reason why Brighton should not be brought to Bermondsey. But steady work and sobriety of life are, in Mr. Ramsay's view, the great panaceas. They hardly rise to the lecturer's admonition to fix the target to the stars, but it is easier to observe them than to procure supplies, from, say, a Metropolitan ozone company, or to get a footing in somebody else's house.

